# Selective and Effective Gold Recovery from Printed Circuit Boards and Gold Slag Using Amino-Acid-Functionalized Cellulose Microspheres

**DOI:** 10.3390/polym15020321

**Published:** 2023-01-08

**Authors:** Fulai Hao, Jifu Du, Lifang Peng, Manman Zhang, Zhen Dong, Yanbai Shen, Long Zhao

**Affiliations:** 1 School of Resources and Civil Engineering, Northeastern University, Shenyang 110819, China; 2 Changchun Gold Research Institute, China National Gold Group Co., Ltd., Changchun 130012, China; 3 School of Nuclear Technology and Chemistry & Biology, Hubei University of Science and Technology, Xianning 437100, China; 4 State Key Laboratory of Advanced Electromagnetic Engineering and Technology, School of Electrical and Electronic Engineering, Huazhong University of Science and Technology, Wuhan 430074, China

**Keywords:** amino acid, cellulose microspheres, Au recovery, selectivity

## Abstract

The hydrometallurgical recovery of gold from electronic waste and gold slag is a hot research topic. To develop a cost-effective and environmentally friendly adsorbent for gold recovery, four types of amino-acid (arginine, histidine, methionine, and cysteine)-functionalized cellulose microspheres were prepared via a radiation technique. The adsorption performance of the amino acid resins toward Au(III) ions was systematically investigated by batch experiments. The amino acid resins could absorb Au(III) ions at a wide pH range. The adsorption process was followed by the pseudo-second-order model and Langmuir model. The theoretical maximum adsorption capacity was calculated as 396.83 mg/g, 769.23 mg/g, 549.45 mg/g, and 636.94 mg/g for ArgR, HisR, MetR, and CysR, respectively. The amino acid resins could effectively and selectively recover trace Au(III) ions from the leaching solutions of printed circuit board and gold slag waste. Lastly, the mechanism underlying amino acid resin’s Au(III) ion recovery capability was investigated by FTIR, XRD, and XPS analyses. This work describes a series of cost-effective gold adsorbents with excellent selectivity and adsorption capacity to boost their practical application.

## 1. Introduction

As a popular precious metal, gold (Au) is widely used in the medical, electronic, and electrical industries due to its special properties, such as excellent electrical conductivity and outstanding corrosion resistance [1]. The contradiction between the growing demand for resources in the Earth’s crust and their limited nature has gradually increased the price of Au. Thus, Au recovery from products utilizing these materials, especially electronic waste and gold slag, has received more and more attention [2]. An appropriate recycling approach can not only retrieve the gold from the e-waste, but also reduce the environmental toxicity of e-waste [3].

There are several technologies for Au recovery using leaching solutions, such as cementation, chemical precipitation, solvent extraction, biosorption, electrolysis, membrane separation, ion exchange, and adsorption [1,4,5]. Among these techniques, adsorption has been considered a promising technology to recover trace precious metals from wastewater due to its low cost, great reusability, and easy operation [6,7].

By now, various adsorbents, such as active carbon [8], multiwalled carbon nanotubes [9], covalent organic frameworks [10], metal–organic frameworks [11], nanoparticles [12,13], melanin-based polymers such polydopamine [14,15], natural polymers (cellulose [16,17], and chitosan [18,19]), among others, have been used for waste remediation. Among them, microcrystalline cellulose (MCC) has many advantages, such as sustainability, hydrophilicity, biodegradability, and easy functionalization, which make it very suitable as an adsorbent substrate [17,20,21,22]. Although MCC has many hydroxyl groups in its chemical structure, the adsorption performance of pure MCC is not satisfactory. So, it is necessary to improve the adsorption property via functionalization. The functional groups for Au(III) ion adsorption are often composed of sulfur and nitrogen atoms such as amine [10], thiourea [20], thiol [23], etc. Dong et al. observed that the maximal adsorption quantities of three novel n-aminomethylpyridine (n-AMP, n = 2, 3, 4)-modified cellulose compounds for Au(III) ions were 1.548, 1.915, and 2.418 mmol/g, respectively [24]. Norasikin and colleagues functionalized lignocellulosic coconut pith (LCP) with 3-aminopropyltriethoxysilane (APS) as a Au(III) ion adsorbent (APS-LCP), which expressed a great adsorption capacity of 261.36 mg/g at 30 °C [25].

In recent years, amino-acid-functionalized materials have attracted much attention because amino acid contains carboxylic acid, amine, and sulfhydryl groups (−COOH, −NH_2_, and −SH), which can be easily conjugated to metal ions. Several researchers reported the adsorption and reduction of Au(III) ions by amino acid [11,26,27]. However, very little investigation has been reported for Au recovery from a practical leaching solution of printed circuit boards (PCBs) and gold slag using amino-acid-functionalized materials.

Radiation-induced grafting polymerization (RIGP) is a powerful technique used to modify polymers because RIGP can generate free radicals and form grafting, which is relevant for subsequent chemical modification. This method can reach a high grafting yield (GY) quickly without heating [21,24]. Then, high functional group density can be obtained through subsequent chemical modification such as the ring-opening reaction.

In this study, four types of amino-acid (arginine, histidine, methionine, and cysteine)-functionalized cellulose compounds were prepared via radiation for Au recovery. The Au(III) ion adsorption performance of amino acid resin using the simulated and practical leaching solutions was systematically investigated.

## 2. Materials and Methods

### 2.1. Materials

The MCC microsphere was supplied by Asahi Kasei Chemicals Corporation (Tokyo, Japan). Chloroauric acid (HAuCl_4_·4H_2_O), glycidyl methacrylate (GMA), and Tween 20 were supplied by Sinopharm Chemical Reagent Co., Ltd. Arginine, histidine, methionine, and cysteine were purchased from Mecklin Chemical Reagent Co., Ltd.

### 2.2. Preparation of Amino Acid Resins

The amino-acid-functionalized cellulose was prepared through two steps. Firstly, GMA was grafted on the MCC microsphere using the pre-irradiation grafting technique as reported in previous works [28,29,30]. First, the MCC microsphere (5.0 g) was vacuum-sealed in PE bags. The samples were irradiated with an absorbed dose of 10 kGy with an EB accelerator under the cooling action of dry ice. The irradiated bag was injected with 50 mL of deoxygenated GMA emulsion solution (30 wt% GMA and 3 wt% Tween 20) and put into a water bath at 50 °C for 2 h. Subsequently, the GMA-grafted microsphere (MCC-g-GMA) was separated and washed with de-ionized water, and then dried to a constant weight. The mass gain was calculated using Equation (1):

(1) $$\mathrm{Mass}\;gain=\frac{m_{g}-m_{0}}{m_{0}^{}}\times 100\%$$

where *m*_0_ and *m*_g_ are the weight of MCC and MCC-g-GMA, respectively.

**Scheme 1:**
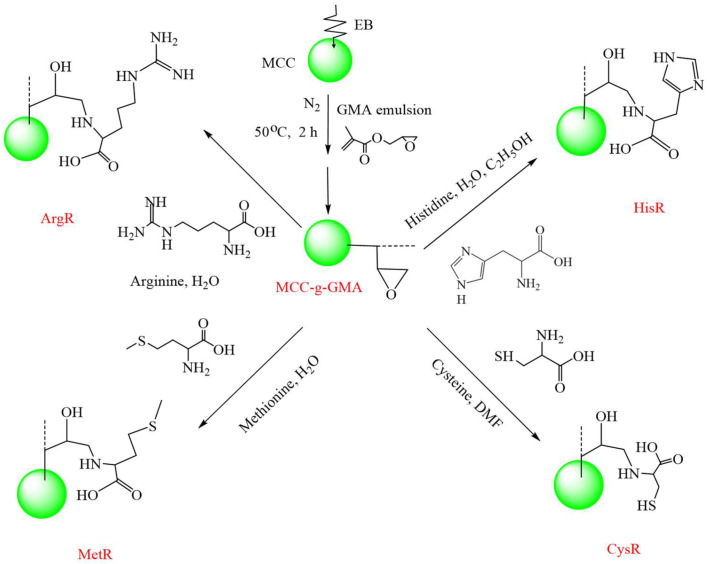
The preparation procedure for the amino acid resins.

Secondly, the MCC-g-GMA (2.0 g) was immersed into different solutions and allowed to react at 80 °C for 24 h. The solution was divided into four types: 20 mL of 10 wt% arginine aqueous solution, 20 mL of 20 wt% cysteine DMF solution, 20 mL of 10 wt% methionine aqueous solution, and 20 mL of 10 wt% histidine 50%H_2_O/50% EtOH solution. After the reaction, these final products were washed with de-ionized water and dried at 60 °C to a constant weight. Scheme 1 shows the preparation procedure for the amino acid resins.

The molar amount of amino acid (MAAA) on the unit amino acid resins was calculated by Equation (2):

(2) $$\mathrm{MAAA}=\frac{m_{r}-m_{g}}{m_{r}\times {\mathrm{Mw}}_{\mathrm{AA}}}$$

where m_g_ and m_r_ are the mass of the MCC-g-GMA and amino acid resins. Mw_AA_ is the molecular weight of a different amino acid.

### 2.3. Characterization

Fourier transform infrared spectroscopy (FTIR) spectra were recorded on an FTIR spectrophotometer (Bruker Tensor 207) in the wavenumber range of 4000–400 cm^−1^. The surface morphology was studied with a scanning electron microscope (SEM, Hitachi SU8000, Japan). The weightlessness curve was recorded on a thermogravimetric analyzer (TGA, TGA 55, TA Instrument mode 600) at temperatures ranging from room temperature to 800 °C at a heating rate of 10 °C/min in a nitrogen atmosphere. An X-ray diffractometer (XRD, Rigaku Corporation, Japan) was applied to capture the pattern images of the materials. The specific surface area of the amino acid resins was measured using the Brunauer–Emmett–Teller method (BET, ASAP2420-4MP Micromeritics, Norcross, GA, USA). The X-ray photoelectron spectroscopy (XPS) spectra were recorded using an AXIS-UItra DLD instrument from Kratos Analytical (Shimadzu, Kyoto, Japan) with a monochromator using Al-Kα radiation.

### 2.4. Adsorption Experiments

A total of 0.01 g of adsorbent was dispersed in 10 mL of Au(III) ion solution. At 30 °C, the glass bottle was shaken in a beaker at 160 rpm. The pH effect was conducted with pH 1–6 and contact time 24 h. The adsorption kinetics were explored at different contact times, from 0.25 to 36 h, at pH 2. The adsorption isotherms were conducted with the initial concentration of 50 to 1000 mg/L and contact time of 24 h. The NaCl effect was investigated at a contact time of 24 h and initial Au(III) ion concentration of 0.1 mmol/L at pH 2. The reuse property was probed at a contact time of 24 h and initial concentration of 100 mg/L at pH 2. The concentration of metal ions was analyzed using the ICP-OES (Agilent 5110, Palo Alto, CA, USA).

The number of Au(III) ions adsorbed by amino acid resins at a certain time (Q_t_) was calculated using Equation (3):

(3) $$Q_{t}=\frac{\left(C_{0}-C_{t}\right)\times V}{m}$$

where C_0_ and C_t_ are the concentration of Au(III) ions before and after adsorption, respectively; Q_e_ represents the adsorption capacity when the adsorption reached equilibrium. V and m are the volume of the solution and the weight of the amino acid resins.

## 3. Results and Discussion

### 3.1. Preparation and Characterization

MCC-g-GMA with a mass gain of 230% was used as the substrate for amino acid modification. Different amino acid resins were obtained after the ring-opening reaction between the epoxy group of GMA and the amine group of amino acid. For the reaction in 20 mL of arginine aqueous solution, 20 mL of cysteine DMF solution, 20 mL of methionine aqueous solution, and 20 mL of histidine 50%H_2_O/50% EtOH solution, the same mass gain, approximately 20%, was obtained.

Considering the molecular weight (174.20, 155, 149.21, and 121.16 for arginine, histidine, methionine, and cysteine), the MAAA of ArgR, HisR, MetR, and CysR was 1.1263, 1.1203, 1.2408, and 1.5011 mmol/g).

**Figure 1:**
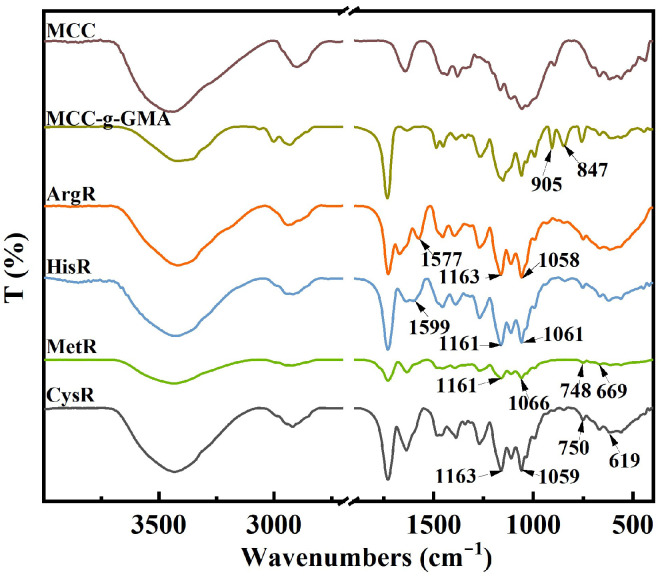
FTIR image of MCC, MCC-g-GMA, and the amino acid resins.

FTIR spectra of MCC, MCC-g-GMA, and the amino acid resins are demonstrated in Figure 1. For MCC-g-GMA, there were new peaks at 905 and 847 cm^−1^, which are the characteristic peaks of epoxy groups [24]. This indicates that GMA was successfully grafted onto MCC. The new characteristic peaks at 1577, 1163, and 1058 cm^−1^ were seen in the spectrum of ArgR, which was related to the vibration of N-H and C-N of arginine [28,31]. In the spectrum of HisR, the vibration adsorption of the N-H (1599 cm^−1^) and C-N (1161 and 1061 cm^−1^) bond were observed. There were two weak peaks at 748 and 669 cm^−1^ in the MetR spectrum, which are characteristic peaks of the C-S bond [32]. In addition, the new peaks at 1161 and 1066 cm^−1^ are characteristic peaks of the C-N bond. For CysR, the characteristic peaks of C-S and C-N bonds at 750, 619 cm^−1^, 1163, and 1059 cm^−1^ were observed. Additionally, the intensity of the characteristic peaks of the epoxide group significantly decreased after amino acid functionalization. So, the synthesis of the amino acid resins was obviously successful.

Figure S1 shows the SEM images of MCC, MCC-g-GMA, and the amino acid resins. It can be seen that the diameter of the modified materials increased, which proves the successful introduction of GMA and amino acids to a certain extent. In addition, Table S1 shows the BET surface areas of materials before and after modification. The BET surface area values of the amino acid resins were too low to prove their porous structures.

### 3.2. Batch Adsorption

#### 3.2.1. Effect of pH

**Figure 2:**
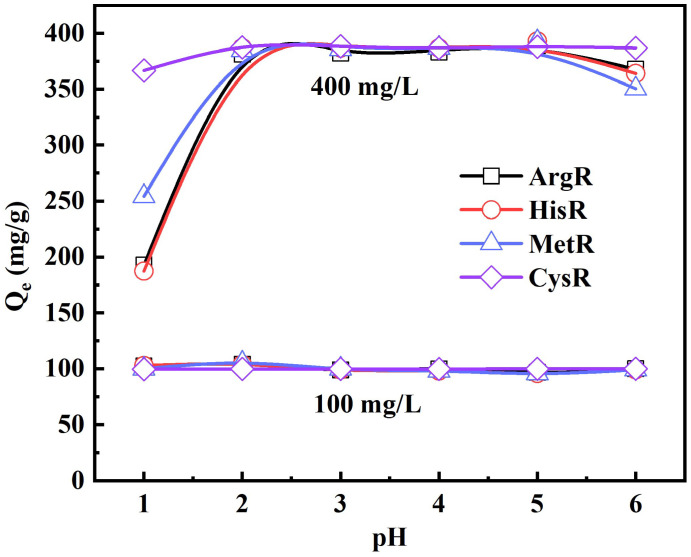
Effect of pH on Au (III) adsorption by amino acid resins (C_0_ = 100 and 400 mg/L, adsorbent mass = 10 mg, volume = 10 mL, T = 30 °C, contact time = 24 h).

The solution pH plays a vital role in the adsorption of metal ions. The influence of pH on the adsorption capacity (*Q*_e_) of amino acid adsorbents at the initial concentrations of 100 and 400 mg/L is shown in Figure 2. At the lower initial concentration of 100 mg/L, the Au (III) ion adsorption capacity of amino acid adsorbents had almost no effect at pH 1–6. The Au(III) ion adsorption capacity at the higher initial concentration of 400 mg/L firstly increased with the increasing pH and then reached a maximum at about pH 2. Under acidic conditions, N atoms on the amino acid adsorbents were protonated to form positive charge centers, which easily attracted AuCl_4_^−^. At pH 1, the concentration of HCl increased, and the increase in the ionic strength and shield effect reduced the number of Au(III) ions adsorbed. With the further increase in pH (pH > 5), the concentration of OH^-^ in the solution increased, and the adsorption competition between OH^-^ and AuCl_4_^-^ also reduced the number of Au(III) ions absorbed. Meanwhile, the Au(III) ion adsorption capacity of non-functionalized MCC and MCC-g-GMA at pH 1–6 is demonstrated in Figure S2, being less than 15 mg/g. This indicates that amino acid functionalization greatly improved the Au(III) ion adsorption capacity.

#### 3.2.2. Adsorption Kinetics: Effect of Adsorption Time

**Figure 3:**
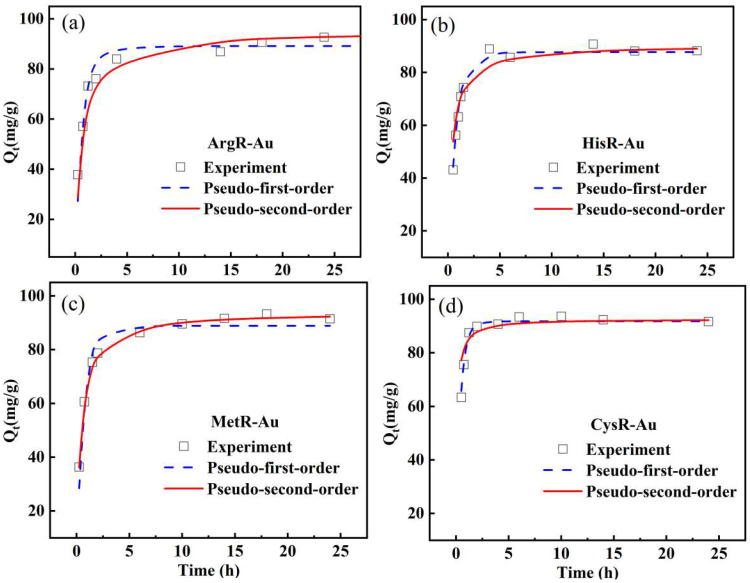
The adsorption kinetics of ArgR (**a**), HisR (**b**), MetR (**c**) and CysR (**d**) for Au(III) ions (C_0_ = 100 mg/L, adsorbent mass = 10 mg, volume = 10 mL, pH = 2, T = 30 °C).

In order to explore the adsorption kinetics, the effect of adsorption time on adsorption capacity was investigated and is shown in Figure 3 (C_0_ = 100 mg/L) and Figure S3 (C_0_ = 400 mg/L). It can be seen that the adsorption capacity increased rapidly initially because there were many active sites on the surface of the adsorbents. With the increase in contact time, the active sites of the adsorbents were occupied continuously, and the adsorption gradually reached equilibrium. The experimental data were fitted by pseudo-first-order (Equation (4)) and pseudo-second-order models (Equation (5)) to evaluate the adsorption kinetics. The initial adsorption rate *h*_0_ (mg/g·min) (*t*→0) is described by Equation (6) [33,34].

(4) $$Q_{t}=Q_{e}(1-e^{-k_{1}t})$$

(5) $$Q_{t}=\frac{k_{2}Q_{e}^{2}t}{1+k_{2}Q_{e}t}$$

(6) $$h_{0}=k_{2}Q_{e}^{2}$$

where *Q_e_* and *Q_t_* (mg/g) are the adsorption mass of Au(III) ions at equilibrium or any time *t*.

The kinetic parameters obtained by fitting are listed in Table 1 (C_0_ = 100 mg/L) and Table S2 (C_0_ = 400 mg/L). The results show that the adsorption of Au (Ⅲ) by the amino acid resins accorded with the pseudo-second-order kinetic model, suggesting the mechanism by which Au(III) ions were absorbed was chemical adsorption [35,36].

#### 3.2.3. Adsorption Isotherms: Effect of Initial Concentration

**Figure 4:**
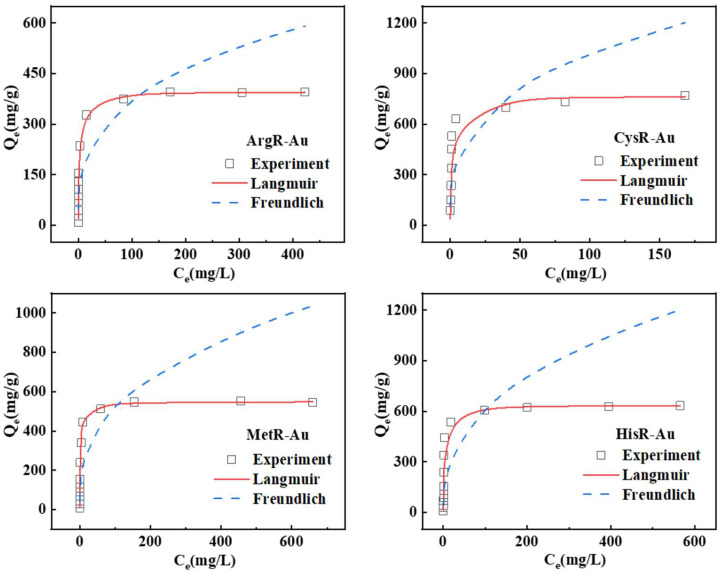
The adsorption isotherms for Au (III) by amino acid resins (adsorbent mass = 10 mg, volume = 10 mL, pH = 2, T = 30 °C, contact time = 24 h).

In order to explore the maximum adsorption capacity of different amino acid resins for Au(III), the adsorption capacity of each amino acid resin under different initial concentrations of Au(III) was studied. As shown in Figure 4, the amount of Au (III) adsorbed on the adsorbents sharply increased with the increase in Au (III) concentration, and then tended to approach a constant value at a higher concentration. The Langmuir (Equation (7)) and Freundlich (Equation (8)) models were used to fit the experimental data [37,38].

(7) $$Q_{e}=\frac{Q_{m}k_{L}C_{e}}{1+(k_{L}C_{e})}$$

(8) $$Q_{e}=k_{F}C_{e}^{1/n_{}}$$

where Q_e_ (mg/g) and C_e_ (mg/L) are the equilibrium adsorption capacity and equilibrium concentration, and Q_m_ (mg/g) is the maximum adsorption capacity.

The adsorption and fitting results are summarized in Table 2. The higher fitting coefficients of the Langmuir model for amino acid resins were greater than 0.99, indicating that the adsorption isotherms of the amino acid resins for Au(III) are more accordance with the Langmuir adsorption isotherm model, and Au(III) ions were absorbed by a monolayer distribution [39]. Theoretically, the maximum Au (III) adsorption capacity of the four amino acid resins is 396.83 mg/g, 769.23 mg/g, 549.45 mg/g, and 636.94 mg/g, suggesting that all amino acid resins are good Au-absorbing materials.

Q_m_ is an important parameter for assessing adsorption performance. Table 3 compares the Au(III) adsorption capacity of the amino acid adsorbents and other adsorbents. As can be seen, the amino acid adsorbents exhibited an excellent adsorption capacity for Au(III).

#### 3.2.4. Thermodynamics: Effect of Temperature on Adsorption

The main thermodynamic parameters in free energy (Δ*G*), enthalpy (Δ*H*), and entropy (Δ*S*) for the Au(III) ion adsorption were determined at 298–318 K. The thermodynamic parameters were determined using the Van’t Hoff equation (Equations (9) to (10)) and are listed in Table 4 [43].

(9) $$\ln K_{d}=\frac{\Delta S}{R}-\frac{\Delta H}{RT}$$

(10) ΔG=ΔH−TΔS

where *K_d_* is the equilibrium constant obtained from *Q_e_*/*C_e_*, *R* is the universal gas constant (8.3144 J/mol × K), and T is the temperature (K).

**Figure 5:**
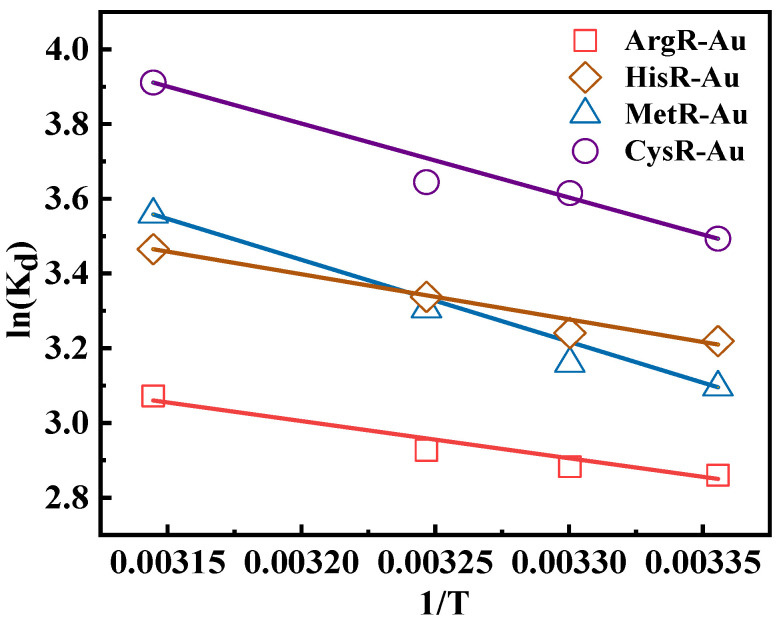
Van’t Hoff plots of amino acid resins for Au(III) ion adsorption (C0 = 100 mg/L, adsorbent mass = 10 mg, volume = 10 mL, pH = 2, contact time = 24 h).

Figure 5 showed Van’t Hoff plots of amino acid resins for Au(III) ion adsorption. The positive value of Δ*S* demonstrated that the adsorption process of Au(III) ions is a random adsorption process at the solid−liquid interface. Δ*G* < 0, Δ*H* > 0 means that the series of adsorption processes are endothermic and indicates a spontaneous reaction [44].

#### 3.2.5. Effect of NaCl

Aqueous metallurgy is a common method of recovering Au ions. After treatment with hydrochloric acid, a large number of Cl^-^ molecules remain. Therefore, the effect of ionic strength on the adsorption performance of an adsorbent must be investigated.

**Figure 6:**
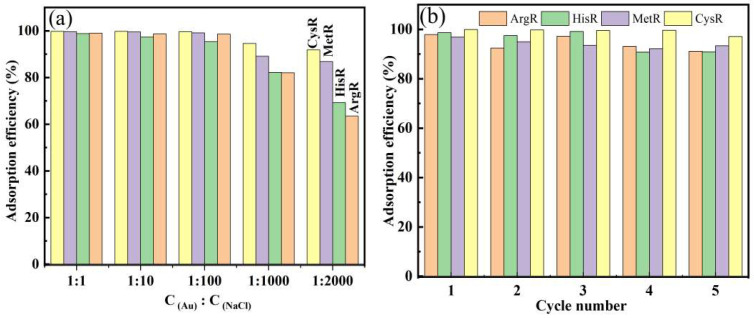
Effect of NaCl on Au(III) ion adsorption by amino acid resins (**a**) and adsorption–desorption process (**b**) (adsorbent mass = 10 mg, pH = 2, V = 10 mL, T = 30 °C, contact time = 24 h).

The effect of NaCl concentration on the Au(III) ion adsorption capacity of amino acid resins is shown in Figure 6a. The results show that the Au (III) adsorption efficiency of amino acid resins decreased with an increase in NaCl concentration. This is mainly because of the shielding effect of chloride ions on the electrostatic interaction between Au(III) ions and the amino acid adsorbents. This proves that the mechanism by which partial Au(III) ions were adsorbed was ion exchange. The adsorption of ArgR-Au and HisR-Au on Au(III) ions was more affected by salt concentration, but when C_(Au)_: C_(NaCl)_ was 1:1000, their adsorption efficiency was more than 80%, and the adsorption effect was also better. For MetR and CysR, although C_(Au)_: C _(NaCl)_ reached 1:2000, their Au(III) ion adsorption efficiency was still more than 85%, indicating that MetR and CysR can effectively adsorb Au (III) ions under high ionic strength. This phenomenon is consistent with the results shown in Figure 2, where it can be seen that the adsorption capacities of MetR and CysR were higher than those of ArgR-Au and HisR at pH 1.

#### 3.2.6. Reuse Property

The reuse property of the adsorbents was also investigated. A total of 1 M thiourea and 1 M HCl mixing solution were used to regenerate the amino acid adsorbents for 24 h. Figure 6b shows the adsorption efficiency after five cycles of the adsorption–desorption process. The adsorption efficiencies of the amino acid adsorbents after five cycles were almost unchanged and greater than 98%. This indicates that the amino acid adsorbents have excellent reusability and practical application prospects.

### 3.3. Adsorption Mechanism

#### 3.3.1. XRD

**Figure 7:**
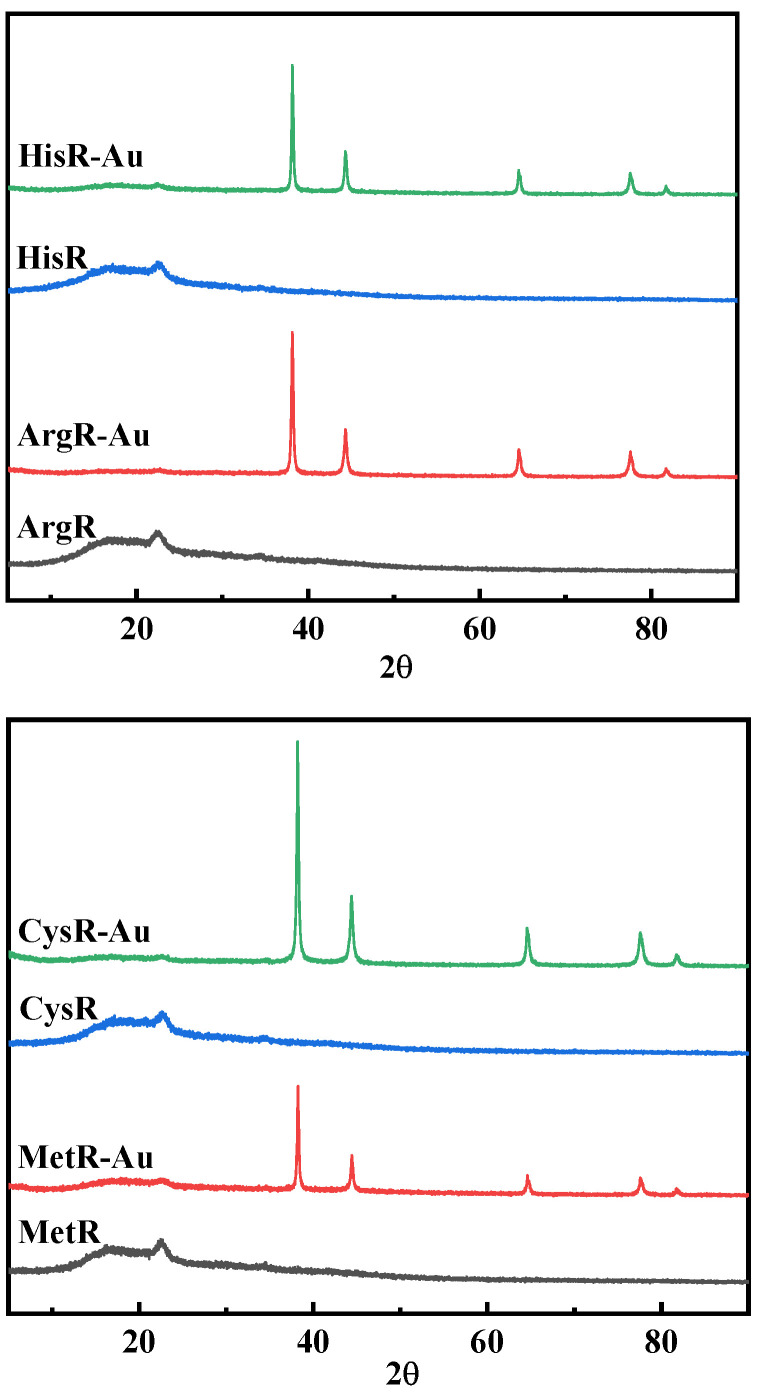
XRD spectra of Au-loaded amino acid resins.

The XRD analysis of amino acid resins before and after Au(III) ion adsorption is shown in Figure 7. The formation of Au (0) after absorption was further confirmed by the XRD spectrum; the new peaks at 38.13°, 44.38°, 64.64°, 77.59°, and 82.11° corresponded to five diffraction peaks at the (111), (200), (220), (311), and (222) crystal faces of Au (0), which appeared after adsorption, suggesting the Au(III) ion recovery was carried out via an adsorption–reduction mechanism [3,24]. 

#### 3.3.2. XPS Analysis

**Figure 8:**
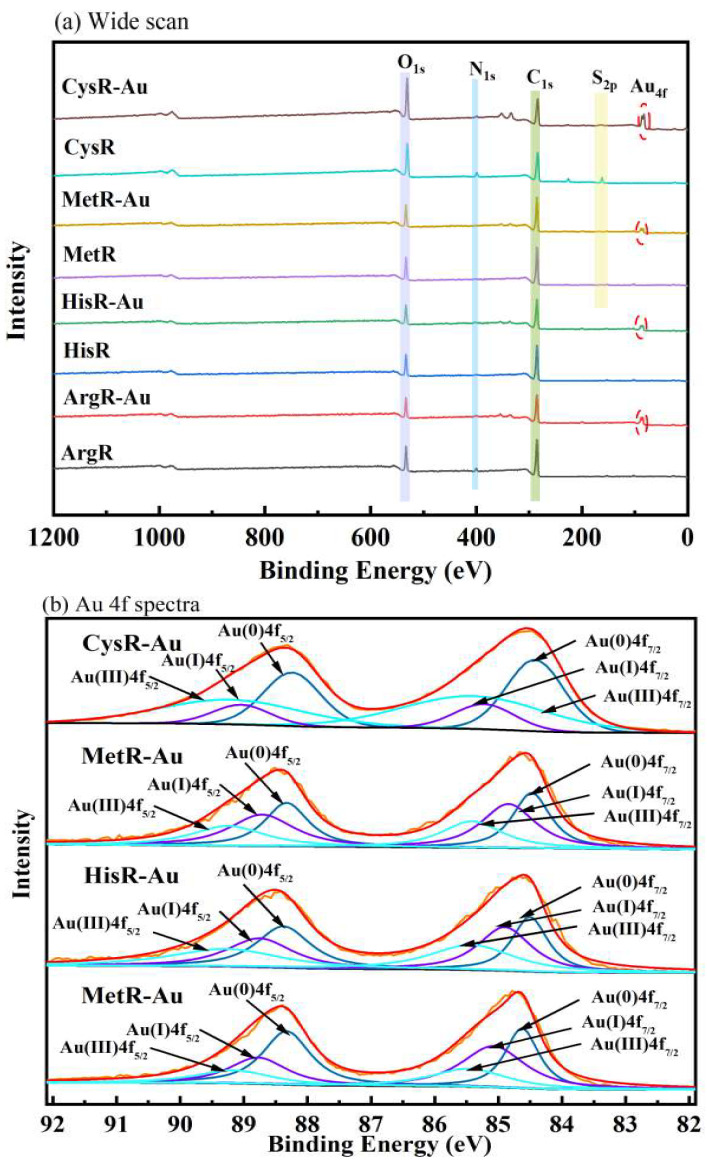
XPS spectra of the amino acid adsorbents before and after adsorption: wide-scan (**a**) and Au4f spectra (**b**).

Figure 8 illustrates the XPS spectra of amino acid resins before and after adsorption. The wide-scan spectra are shown in Figure 8a. The dominant peaks of ArgR and HisR were C1s, O1s, and N1s, and the dominant peaks of MetR and CysR were C1s, O1s, N1s, and S2p, indicating that the four amino acids were successfully introduced into cellulose., The Au4f peak at 82–92 eV appearing after adsorption represents the adsorption of Au (III) by amino acid resins [3].

Figure 8b shows the Au high-resolution XPS spectra. Both the Au4f7/2 and Au 4f5/2 peaks could be fitted to three peaks. After adsorption, the Au (0) and Au (I) signal appeared, demonstrating that Au(III) ions were reduced to Au (I) and Au (0). This result agrees with the XRD findings [45]. Combined with the XPS and XRD results, this shows that the mechanisms by which Au was absorbed on amino acid resins were adsorption–reduction and chelation.

#### 3.3.3. FTIR and EDX

EDX images and FT-IR spectroscopy before and after Au(III) adsorption are shown in Figures S4–S8. It can be seen from FT-IR spectroscopy that the peak positions (C-N and C-S) shifted after gold adsorption, indicating that groups containing N and S of amino acid resins interacted with Au(III). Meanwhile, the EDX images show that a lot of Au(III) was adsorbed on the surface of the resins.

**Scheme 2:**
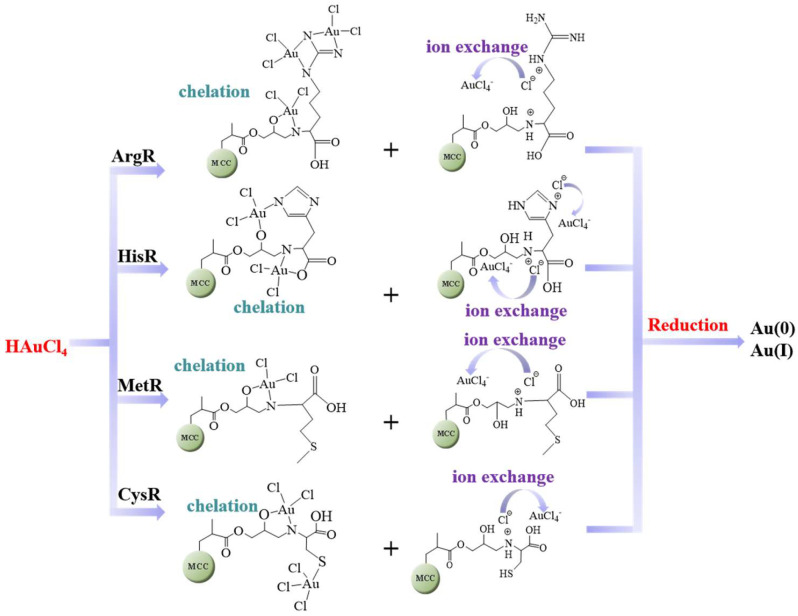
Mechanism of Au(III) ion adsorption onto the amino acid resins.

On the basis of the above analysis, the mechanism by which Au(III) was absorbed on the amino acid resins is proposed in Scheme 2. The functional atoms on the surface of the adsorbent interacted with Au(III) by ion exchange, chelation, and oxidation–reduction reactions.

### 3.4. Practical Application of Au(III) Ion Recovery from Leaching Solutions

**Scheme 3:**
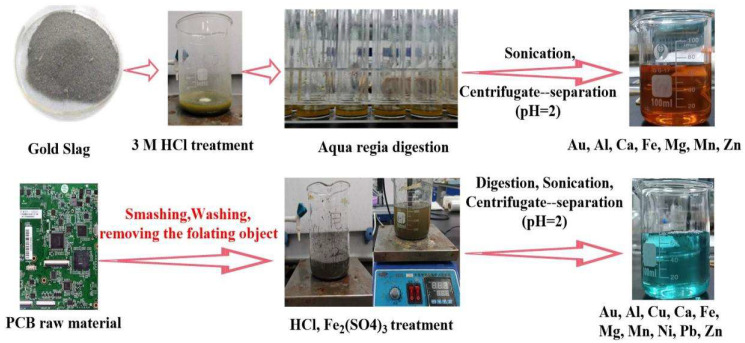
The leaching process of slag and PCB.

The recovery of gold from the leaching solution of PCB and gold slag waste is considered a potential gold resource. However, these acidic solutions also contain other base metals, such as Cu, Ni, Fe, and Zn. [10,46,47]. Therefore, it is very important that the selective recovery of trace Au(III) ions (µg/L) from practical leaching solution be investigated. In the current work, the practical application of amino acid adsorbents for Au(III) ion recovery from two practical leaching solutions, gold slag and PCB, was conducted. The leaching solution of gold slag and PCBs was prepared via the acid treatment procedure shown in Scheme 3.

#### 3.4.1. Gold Recovery from Gold Slag Leaching Solutions

The gold slag was obtained from the tailings of a pure quartz gold ore in Xinjiang, China. In the leaching process, gold slag with a mass of 20 g was used as a raw material, into which 3 M HCl solution was slowly added. After filtering, the solid residue obtained after the above treatment was digested with 80 mL of aqua regia (12 M HCl: 14 M HNO_3_ = 3:1) for 11 cycles. Then, the solid residues were transferred into a beaker containing de-ionized water, sonicated, separated via centrifugation, and filtered. Thus, the slag leaching solution was obtained.

For the adsorption experiment, 0.02 g of amino acid resins was soaked in 5 mL of gold slag leaching solution (pH 2). The composition of the gold slag leaching solution before and after adsorption is listed in Table 5. The other metals present were Au, Al, Ca, Fe, Mg, Mn, and Zn. Figure 8a shows the adsorption efficiency of amino acid adsorbents for Au(III) ions in the presence of other metal ions. The Au(III) ion recovery efficiencies of ArgR, HisR, MetR, and CysR were 68.1%,75.1%, 82.1%, and 74.1%, respectively.

#### 3.4.2. Gold Recovery from PCB Leaching Solutions

**Figure 9:**
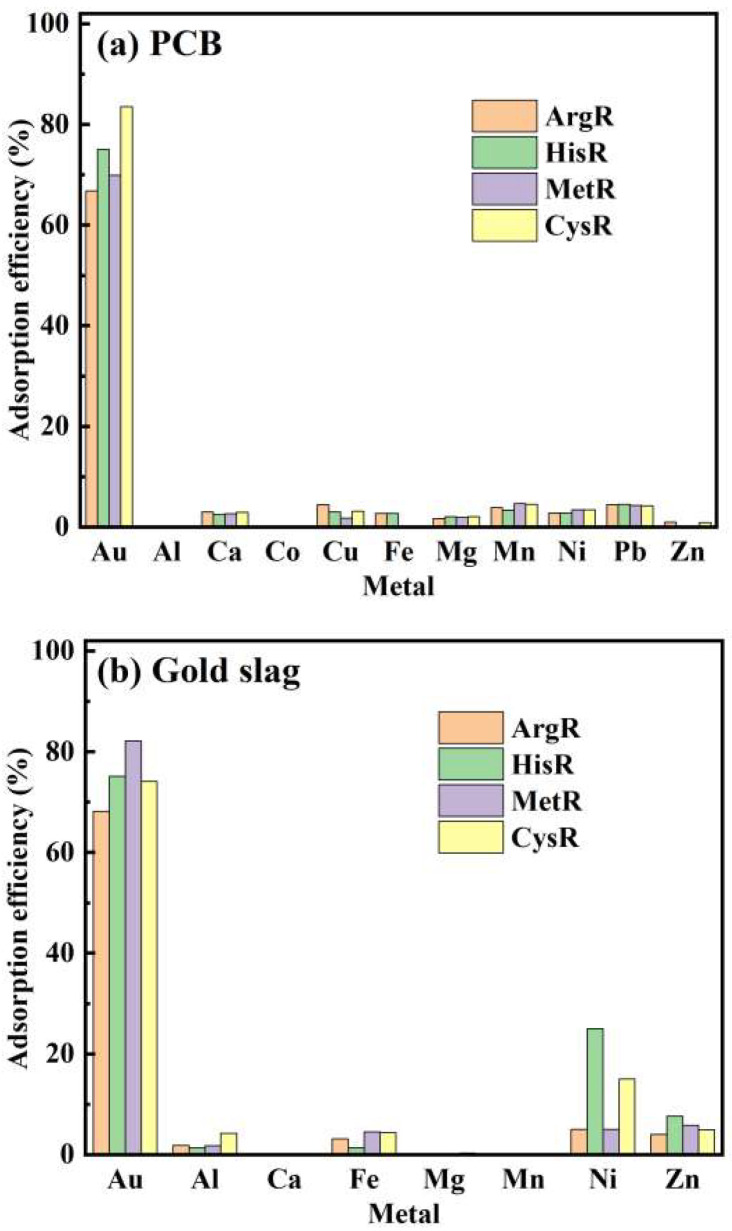
The adsorption efficiency of amino acid resins for different metals.

For the printed circuit boards (PCBs), 20 g of PCB was used as a raw material. First, a PCB was smashed, and the objects floating in the water were removed. Then, the PCB was mixed with 3 M HCl for 3 h and 3% FeSO_4_ for 8 h. The solid residues were treated with a similar process to that used for the slag residues to obtain the PCB leaching solution. The main metals present were Au, Al, Cu, Ca, Fe, Mg, Mn, Ni, Pb, and Zn. Subsequently, 0.02 g of amino acid resin was soaked in 5 mL of PCB leaching solution (pH 2). Figure 9 show the adsorption efficiency of different amino acid adsorbents for Au(III) ions in the presence of other metal ions. The Au(III) ion recovery efficiencies of ArgR, HisR, MetR, and CysR were 66.8%, 75.1%, 69.9%, and 83.5%, respectively.

Despite the existence of excessive co-leached metal ions, amino acid resins also showed a remarkable selectivity for Au(III) ions. This is mainly attributed to the presence of Au(III) ions as an anion ([AuCl_4_]^−^ and [AuCl_3_(OH)]^−^) at pH 1–6. In contrast, the other competitive common metal ions existed as cations, which exhibited electrostatic repulsion to the positively charged amino acid resins. In addition, Au is a soft metal that has a higher affinity for donor atoms such as N and S atoms on amino acid resins. In conclusion, the amino acid adsorbents rarely captured any common metals except for Au(III) ions, indicating that the amino acid adsorbents possess potential for use in gold resource recovery from PCBs and gold slag.

## 4. Conclusions

Four types of amino-acid-functionalized cellulose adsorbents for Au recovery were successfully prepared by EB radiation grafting and further modification. The amino acid resins could absorb Au(III) ions at a wide pH range. The adsorption isotherms were well fitted by the Langmuir model, and the maximum adsorption capacities of the four amino acid resins for Au(III) were 396.83 mg/g, 769.23 mg/g, 549.45 mg/g, and 636.94 mg/g. The adsorption mechanisms investigated by XRD and XPS analyses were adsorption–reduction and chelation by N and S atoms. The adsorption–desorption experiments showed that amino acid resins can be efficiently regenerated by 1 M thiourea and 1 M HCl for further Au(III) ion adsorption. The application of amino acid resins in practical leaching solutions showed that amino acid resins can effectively and selectively recover Au from practical PCB and gold slag leaching solutions.

## Figures and Tables

**Table 1:** Kinetic parameters obtained from kinetic models.

Adsorbents	Pseudo-First-Order Model			Pseudo-Second-Order Model			
	k_1_ (h^−1^)	Q_e_ (mg/g)	R^2^	k_2_ (g/(mg·h))	Q_e_ (mg/g)	R^2^	h_0_
ArgR	1.4596	89.1507	0.9098	0.0182	95.0570	0.9995	164.45
HisR	1.5424	88.8839	0.9427	0.0268	93.8086	0.9997	235.84
MetR	1.4036	87.6844	0.9533	0.0327	90.2527	0.9995	266.36
CysR	2.5200	91.7400	0.9809	0.1080	92.5926	0.9998	925.96

**Table 2:** Adsorption isotherm parameters for Au(III) absorbed by amino acid resins.

Adsorbents	Langmuir			Freundlich		
	Q_m_ (mg/g)	K_L_(L/mg)	R^2^	K_F_ (mg/g)	n	R^2^
ArgR	396.8	0.4903	0.9999	92.1751	3.2527	0.6644
HisR	636.9	0.3668	0.9999	114.5304	2.6953	0.7217
MetR	549.5	0.5741	0.9999	112.2277	2.9200	0.7470
CysR	769.2	0.7927	0.9993	262.4000	3.3653	0.7364

**Table 3:** Comparison of adsorption capacity of amino acid adsorbents with other available adsorbents.

Adsorbents	pH	T (°C)	Qm (mg/g)	Ref.
2-AMPR	2.4 ± 0.2	-	537.613	[24]
DACS-TA	2.0	35	298.5	[40]
Zr-MOF	2.0	-	374.866	[41]
Ni_0.6_Fe_2.4_O_4_ nanoparticels	4.0	25	383.9	[42]
APS-LCP	4.0	30	261.36	[25]
ArgR	2.0	30	396.8	This work
HisR	2.0	30	636.9	This work
MetR	2.0	30	549.5	This work
CysR	2.0	30	769.2	This work

**Table 4:** Thermodynamic parameters of the adsorption of Au(III) ions on amino acid resins.

Adsorbents	Δ*H* (KJ/mol)	Δ*S* (J/mol/K)	Δ*G* (KJ/mol)				R^2^
			298 K	303 K	308 K	318 K	
ArgR	8.5453	52.2784	−7.0337	−7.2981	−7.5564	−8.0792	0.9460
HisR	10.1997	60.8359	−7.9293	−8.2336	−8.5378	−9.1461	0.9695
MetR	18.8498	88.7484	−7.6270	−8.0410	−8.4847	−9.3722	0.9827
CysR	15.9659	82.5559	−8.6358	−9.0485	−9.4613	−10.2869	0.9619

**Table 5:** The composition of leaching solution before and after ArgR, HisR, MetR, and CysR adsorption.

Element	Raffinate (mg/L)									
	PCB					Au Slag				
	Feed (mg/L)	ArgR	HisR	MetR	CysR	Feed (mg/L)	ArgR	HisR	MetR	CysR
Au	0.449	0.149	0.112	0.135	0.074	0.386	0.123	0.096	0.069	0.1
Al	108.6	108.4	108.5	108.5	107.6	351.9	345.4	347.2	345.6	336.9
Ca	252.8	245.2	246.5	246.1	245.4	261.9	264.2	262.9	264.0	261.5
Co	0.229	0.223	0.225	0.226	0.229	---	---	---	---	---
Cu	3669	3506	3559	3603	3555	---	---	---	---	---
Fe	0.37	0.36	0.36	0.38	0.38	114.7	111.1	113.1	109.5	109.7
Mg	7.530	7.403	7.379	7.385	7.376	158.6	158.6	160.6	160.2	158.1
Mn	0.530	0.509	0.512	0.505	0.506	13.89	13.83	13.76	13.73	13.64
Ni	25.38	24.68	24.68	24.51	25.36	---	---	---	---	---
Pb	35.86	34.27	34.24	34.33	34.34	---	---	---	---	---
Zn	367.8	364.3	367.1	368.8	364.5	1.232	1.352	1.081	1.299	1.218

## Data Availability

Not applicable.

## Supplementary Materials

The following supporting information can be downloaded at: https://www.mdpi.com/article/10.3390/polym15020321/s1, Figure S1: SEM images of MCC, MCC-g-GMA and the amino acid resins; Figure S2: Effect of pH on Au (Ⅲ) adsorption by MCC and MCC-g-GMA; Figure S3: The adsorption kinetics of amino acid resins for Au(III) ions; Figure S4: FTIR spectra of ArgR, HisR, MetR and CysR; Figure S5: EDX images of ArgR after Au (Ⅲ) adsorption; Figure S6: EDX images of HisR after Au (Ⅲ) adsorption; Figure S7: EDX images of MetR after Au (Ⅲ) adsorption; Figure S8: EDX images of CysR after Au (Ⅲ) adsorption; Table S1: BET surface areas of MCC, MCC-g-GMA and the amino acid resins; Table S2: Kinetic parameters and correlation coefficients (R2) of the amino acid resins.
