# Supplementary material for: Selective and Effective Gold Recovery from Printed Circuit Boards and Gold Slag Using Amino-Acid-Functionalized Cellulose Microspheres
Source: Polymers (Basel). 2023 Jan 8;15(2):321. doi: 10.3390/polym15020321 (PMC9863566; doi:10.3390/polym15020321)
Supplement: Supplementary file 1 [file polymers-15-00321-s001.zip › polymers-2059188-supplementary.pdf]

Supplementary material for

Selective and Effective Gold Recovery from Printed Circuit Boards and Gold Slag by Amino Acid Functionalized Cellulose Microspheres

Fulai Hao[1,3], Jifu Du[2*], Lifang Peng[4], Manman Zhang[4],

Zhen Dong[4], Yanbai Shen[1*], Long Zhao[4*]

[1]School of Resources and Civil Engineering, Northeastern University, Shenyang 110819, China
[2]School of Nuclear Technology and Chemistry & Biology, Hubei University of Science and Technology, Xianning 437100, China
[3]Changchun Gold Research Institute, China National Gold Group Co. Ltd, Changchun 130000, China
[4]State Key Laboratory of Advanced Electromagnetic Engineering and Technology, School of Electrical and Electronic Engineering, Huazhong University of Science and Technology, Wuhan 430074, China.

[*]Correspondence: duzidedu@163.com (Jifu Du); shenyanbai@mail.neu.edu.cn (Yanbai Shen); zhaolong@hust.edu.cn (Long Zhao)
Correspondence: author at: State Key Laboratory of Advanced Electromagnetic Engineering and Technology, School of Electrical and Electronic Engineering, Huazhong University of Science and Technology, Wuhan 430074, China

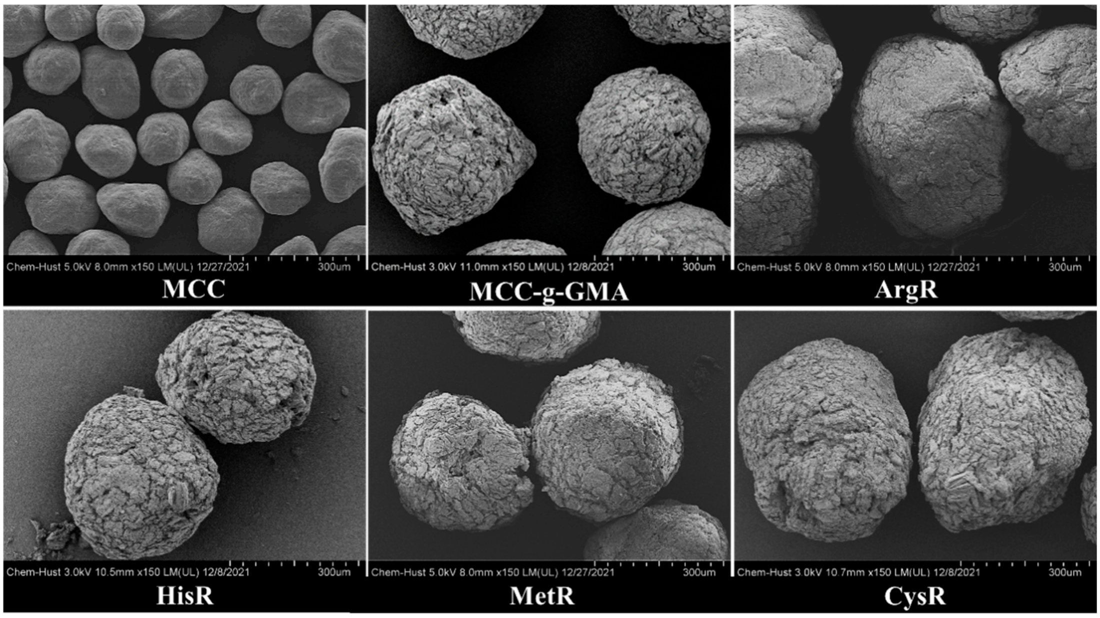

Figure S1 SEM images of MCC, MCC-g-GMA and the amino acid resins

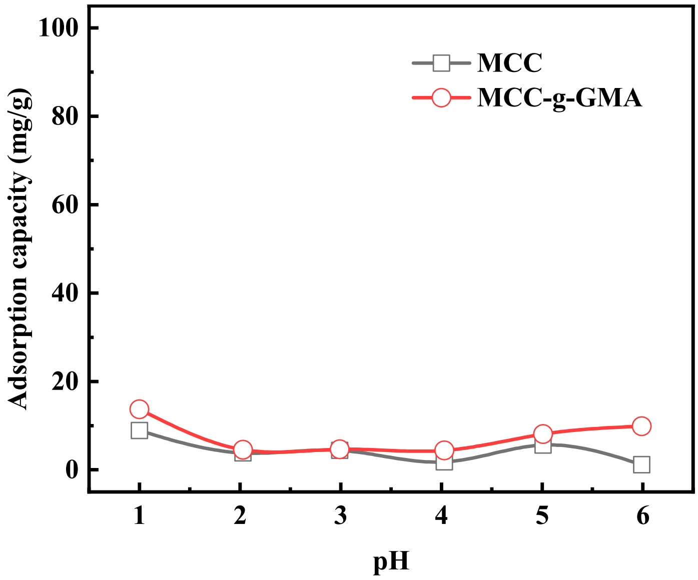

Figure S2 Effect of pH on Au (III) adsorption by MCC and MCC-g-GMA. (C_0=100 mg/L, adsorbent mass=10 mg, volume=10 mL, T=30 °C).

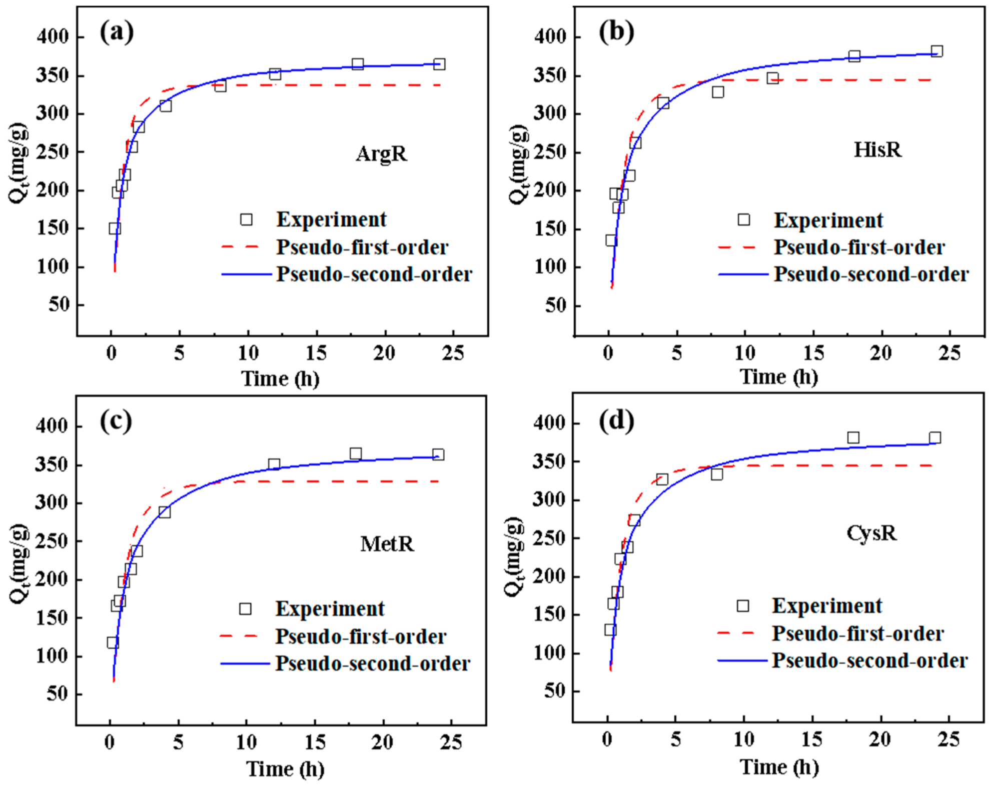

Figure S3 The adsorption kinetics of amino acid resins for Au(III) ions. (C_0=400 mg/L, adsorbent mass=10 mg, volume=10 mL, pH=2, T=30 ℃).

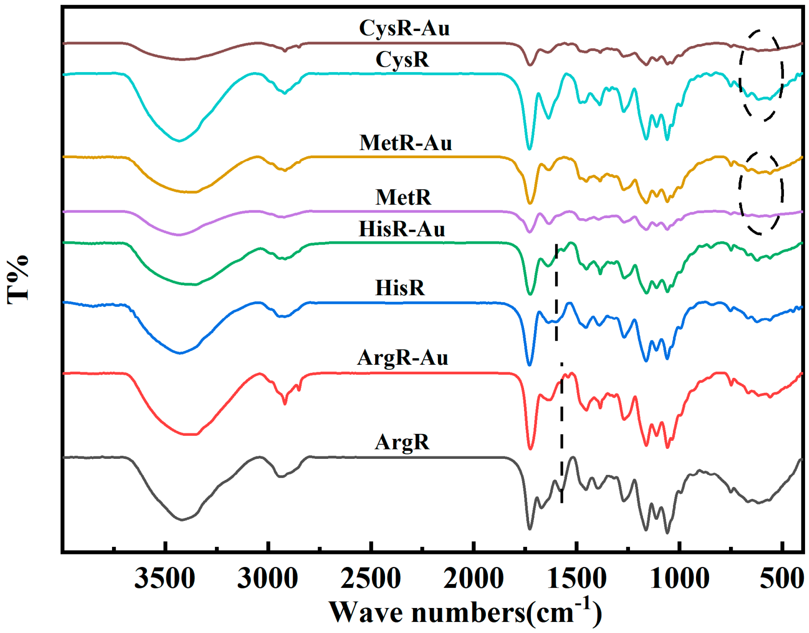

Figure S4 FTIR spectra of ArgR, HisR, MetR and CysR before and after the adsorption of Au(III)

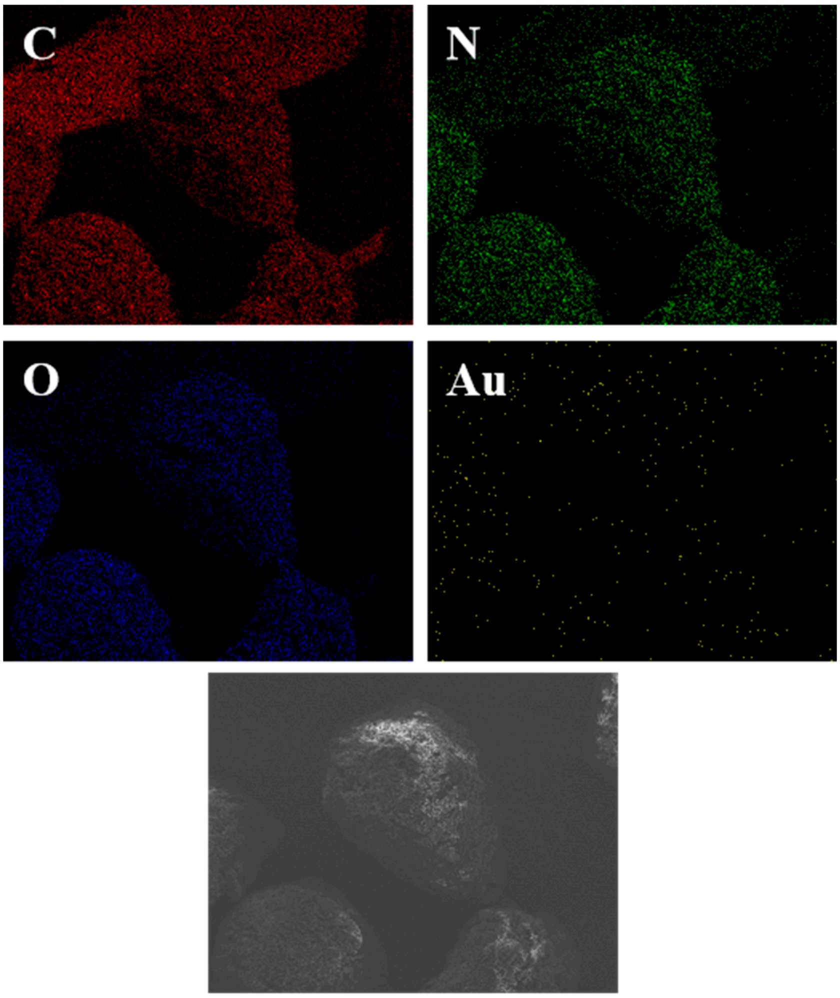

Figure S5 EDX images of ArgR after Au (Ⅲ) adsorption

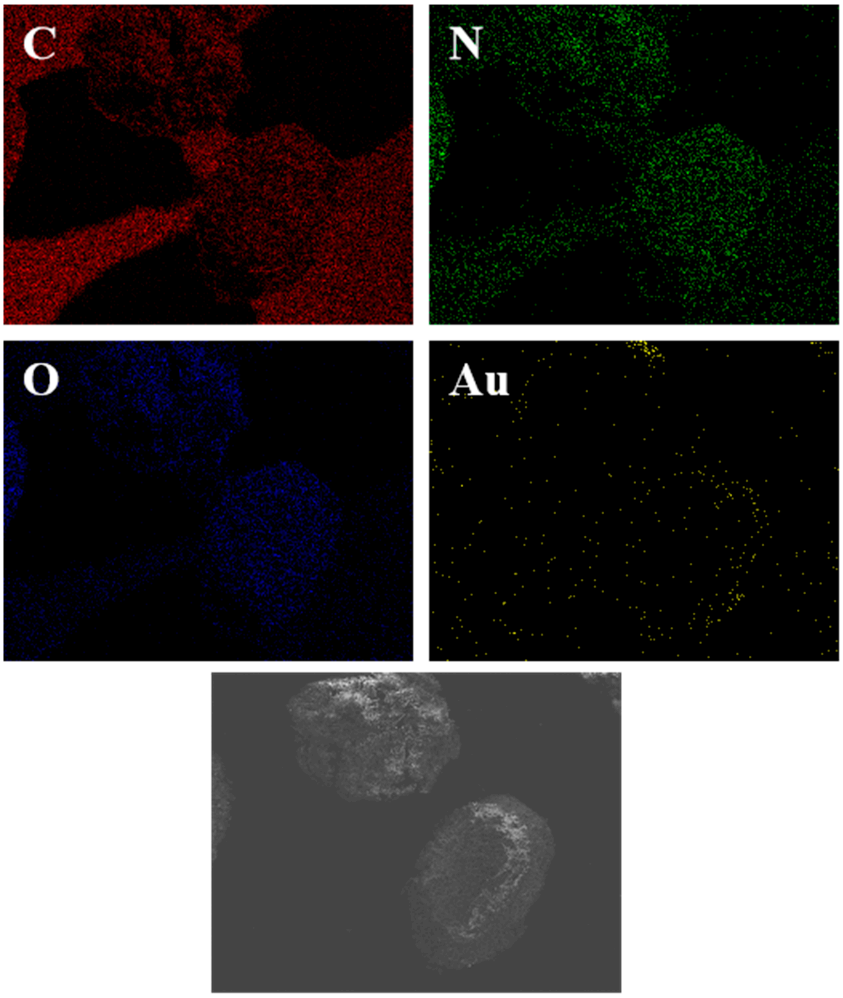

Figure S6 EDX images of HisR after Au (Ⅲ) adsorption

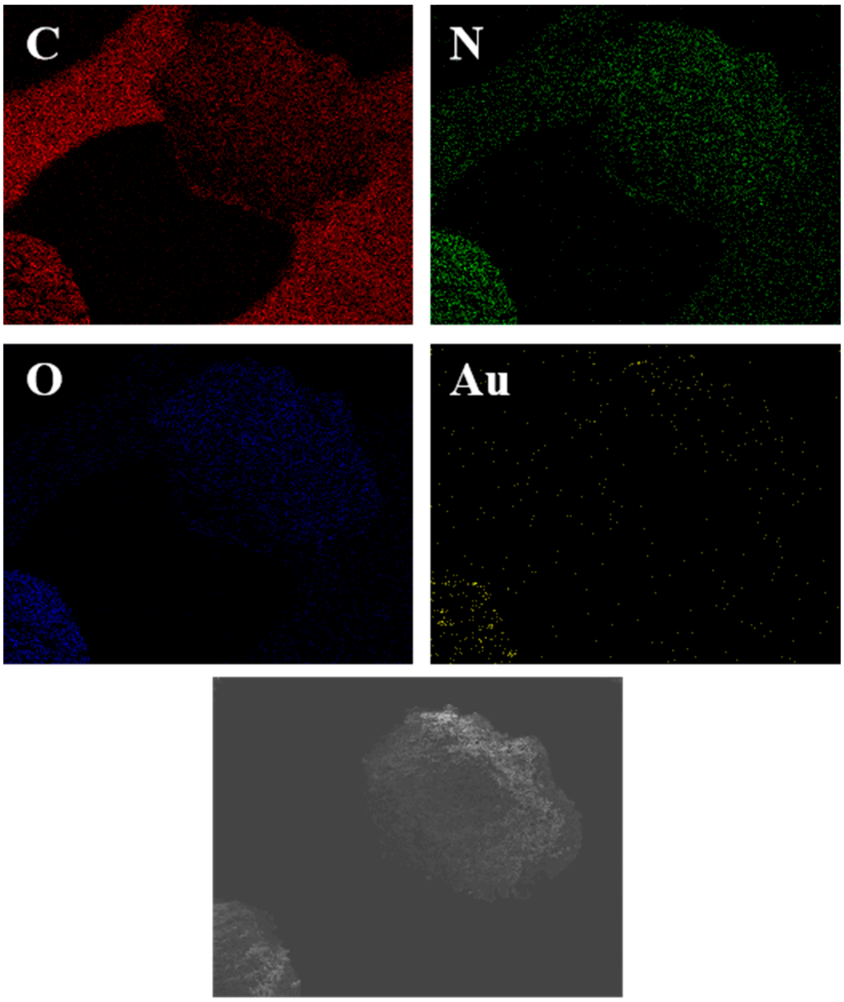

Figure S7 EDX images of MetR after Au (III) adsorption

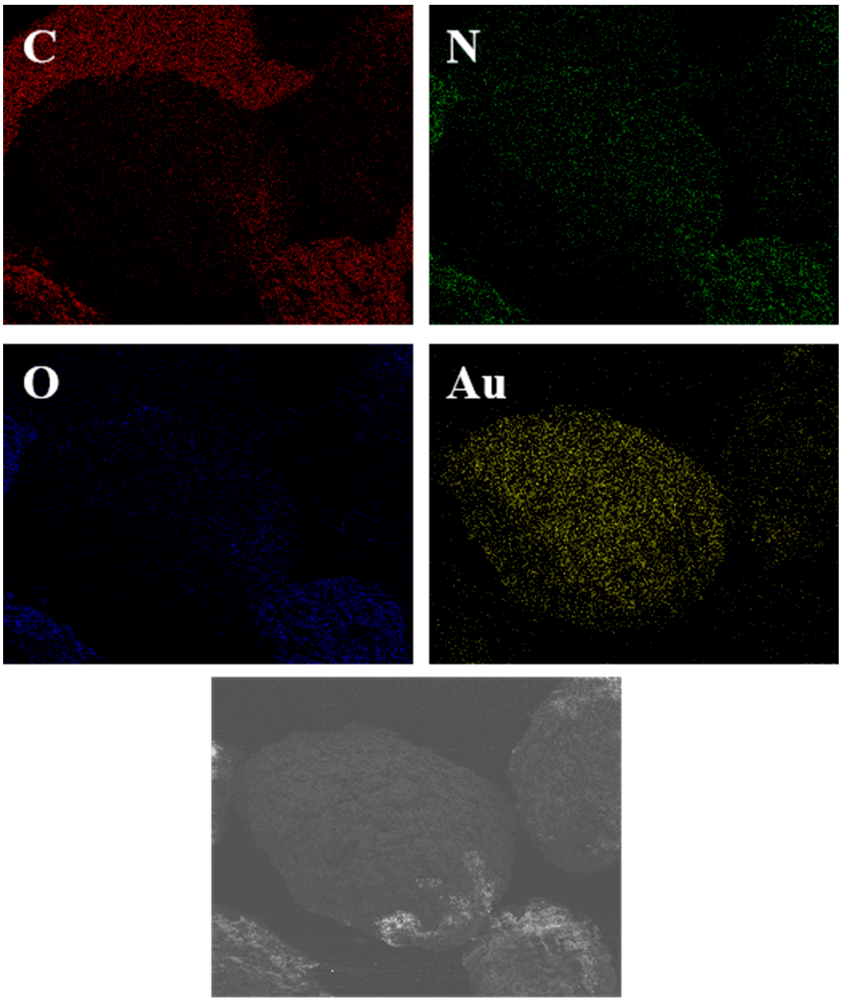

Figure S8 EDX images of CysR after Au (Ⅲ) adsorption

Table S1 BET surface areas of MCC, MCC-g-GMA and the amino acid resins.

Material	BET surface (m^2/g)
MCC	0.4908
MCC-g-GMA	1.0144
ArgR	1.0482
HisR	1.8206
MetR	1.7532
CysR	1.0548

Table S2 Kinetic parameters and correlation coefficients (R^2) of the amino acid resins. (C_0=400 mg/L, adsorbent mass=10 mg, volume=10 mL, pH=2, T=30 ℃)

Adsorbent	Pseudo-first-order			Pseudo- second-order		
	$k_1(h^{-1})$	Qe(mg/g)	R^2	k_2(g/(mg·h))	Qe(mg/g)	R^2
**ArgR**	1.2979	337.8255	0.8356	0.0042	374.5318	0.9996
**HisR**	0.9543	344.8416	0.8083	0.0026	393.7008	0.9976
**MetR**	0.9187	328.5267	0.8092	0.0026	375.9398	0.9965
**CysR**	1.0212	345.0596	0.8846	0.0029	387.5969	0.9944
